# Use of multikinase inhibitors/lenvatinib in patients with synchronous/metachronous cancers coinciding with radioactive‐resistant differentiated thyroid cancer

**DOI:** 10.1002/cam4.5107

**Published:** 2022-10-06

**Authors:** Marcel Sambo

**Affiliations:** ^1^ Endocrinology and Nutrition Department General University Hospital Gregorio Maranon Madrid Spain

**Keywords:** differentiated thyroid carcinoma, multikinase inhibitors, multiple primary malignant neoplasms, synchronous/metachronous cancers

## Abstract

This review focuses on patients with differentiated thyroid carcinoma (DTC) associated with multiple primary malignant neoplasm (MPMN) treated by multikinase inhibitors (MKIs) as systemic treatment for advanced disease. Despite the increasing frequency of MPMNs (many at an advanced stage) and the usefulness of MKIs for multiple metastatic cancers, published data on the management of MPMN and MKI therapies in this scenario are scarce. There are infrequent descriptions of patients with advanced MPMN treated with MKIs, but only a few have described advanced DTC. The management of MPMNs, including DTC and its particular circumstances, is reviewed, focusing on the evidence for MKI therapies. Some considerations for MPMN patients with advanced DTC are discussed, with the intention of helping physicians make decisions in these challenging situations and improving treatment and patient outcomes.

## Abstract 2

Although the frequency of multiple primary malignant neoplasms (MPMNs) is increasing, published data on the management of MPMNs with multikinase therapies are scarce, particularly for advanced differentiated thyroid carcinoma (DTC). This article reviews the management of MPMNs, including considerations for MPMN patients with advanced DTC.

## 1 INTRODUCTION AND HISTORICAL PERSPECTIVE

The entity of multiple neoplasms is not rare or new, having been first described by Billroth in 1889^1^ and further reported on by Owen^2^ in 1921.

Multiple primary malignant neoplasm (MPMN) is defined as more than one primary histologically distinct malignant tumor that occurs in a single individual and may include solid cancers and hematologic malignancies. MPMN can be divided into two categories (based on International Association of Cancer Registries and International Agency for Research on Cancer definitions): (i) synchronous — malignancies occurring within 6 months of a previous malignant neoplasm; and (ii) metachronous — defined as malignancies occurring more than 6 months apart.^3^

The prevalence of MPMN is reported to be 2%–17%.^3^, ^4^, ^5^ It is expected that the prevalence of MPMN (mainly metachronous) will increase in the future due to increasingly aging populations combined with improved cancer survival, improved diagnostic tests, increasingly sophisticated treatment, better screening, and enhanced surveillance of patients with previous cancer.^3^

According to the National Cancer Institute's Surveillance, Epidemiology and End Results Program, cancers with the best survival rates are breast, Hodgkin's lymphoma, melanoma, prostate, testicular, and differentiated thyroid cancer (DCT), which all have a 5‐year survival rate of >85% (the 10‐year survival rate for papillary thyroid carcinoma [PTC] is 95–99%).^6^ Because of the high cure rates, most of these patients will survive having cancer, and will therefore be at increased risk for development of a second primary cancer (SPC), with a lifetime risk as high as 37%.^4^, ^5^

DTC is the most common endocrine malignancy, accounting for approximately 0.5%–1.5% of all cases (in adulthood and childhood) and more than 90% of all the thyroid cancers and malignancies of the endocrine system. The most common DTC is PTC. DTC incidence is increasing worldwide, mainly due to PTC.^7^, ^8^ So, as expected, due to the rising increasing incidence and low disease‐related mortality rate, MPMNs are not rare in patients with DTC.^9^

## 2 CURRENT SITUATION

Studies report that patients with DTC have an associated 6%–39% higher risk of SPCs than the general population (greater in younger patients) and this has been shown to have a negative impact on prognosis.^7^, ^10^, ^11^, ^12^, ^13^, ^14^, ^15^, ^16^ It has been shown that there is an increased incidence of multiple locations for SPCs both before, during, and after a diagnosis of DTC.^8^, ^9^, ^12^, ^13^, ^14^, ^17^, ^18^

There appears to be an increased and persistent two‐way association, verified by updated large epidemiology studies and a meta‐analysis of breast (the most frequent correlated tumor), kidney (renal cell carcinoma [RCC]) and stomach/gastric cancer in DTC patients. Reciprocal association has also been evidenced with melanoma, colon, prostate, scrotum, ovarian, brain, central nervous system cancer, and leukemia, irrespective of which tumor occurred first.^7^, ^8^, ^9^, ^10^, ^12^, ^13^, ^16^, ^17^, ^18^, ^19^, ^20^, ^21^, ^22^, ^23^

A number of factors, including (i) endogenous such as inherited/genetic predisposition (whether or not as part of specific syndromes) (Table 1, ^24^, ^25^, ^26^, ^27^), abnormal embryo development, immune‐associated diseases and/or comorbidities affecting carcinogen sensitivity; (ii) behavioral or lifestyle influences and environmental exposures; (iii) surveillance bias, or late iatrogenic effects of therapies for DTC/other primary tumors, could explain the enhanced general SPC risk and, particularly, in DTC patients.^3^, ^4^, ^15^, ^18^

**TABLE 1:** (A) Genetic DTC predisposition syndromes associated with an increased risk of developing MPMN, and (B) common driver mutations in non‐medullary thyroid cancer.^24^, ^25^, ^26^, ^27^

(A) Genetic DTC predisposition syndromes	(B) Mutations in non‐medullary thyroid cancer					
	Mutated/altered gene(s)	Relevant thyroid cancer histotypes	Mutated/altered gene(s)	Relevant thyroid cancer histotypes	Mutated/altered gene(s)	Relevant thyroid cancer histotypes
FAP and Gardner syndrome	*RET‐PTC* fusions	PTC PDTC	*TP53*	PDTC ATC	*PTEN*	PDTC ATC
PTEN‐hamartoma tumor syndrome/Cowden disease	*BRAF (*generally *V600E/K* mutations)	PTC PDTC ATC	*RAS*	PTC FTC PDTC ATC	*EGFR*	PTC PDTC ATC
Peutz‐Jeghers syndrome	*PAX8‐PPARG (PPARγ)* fusions	FTC			*P13K*	FTC PDTC ATC
Pendred syndrome	*NTRK* rearrangement	PTC	*TERT*	PTC FTC HCC PDTC ATC		
Carney complex	*AXIN1*	ATC				
Werner syndrome	*CTNNB1*	PDTC ATC			*EIF1AX*	PDTC ATC
Birt‐Hogg‐Dube syndrome	*CTNNB4*	ATC				
Dicer1 syndrome	*FLT3*	PDTC	*APC*	PDTC	*SMAD4*	PDTC
	*ATM*	PDTC	*ALK*	PDTC ATC	*ERBB4/HER4*	PDTC
	*KIT*	PDTC				

Abbreviations: ATC, anaplastic thyroid carcinoma; FAP, familial adenomatous polyposis; FTC, follicular thyroid carcinoma; HCC, Hürthle cell carcinoma; PTC, papillary thyroid carcinoma; PDTC, poorly differentiated thyroid carcinoma.

Specifically, the association of radioactive iodine (RAI) therapy and risk of SPCs following DTC remains widely debated.^12^ This association has suggested an enhanced SPC risk for both solid tumors and leukemia in patients with RAI‐treated DTC compared with DTC survivors not exposed to RAI, mainly in younger patients. This risk significantly increases linearly with each increment of cumulative iodine‐131 (I^131^).^10^, ^15^, ^16^, ^18^, ^19^, ^28^, ^29^, ^30^ However, this correlation has not been fully confirmed.^8^, ^31^, ^32^, ^33^

Some studies have also found an increased risk for SPC (including DTC) after external beam radiation therapy (EBRT) for other primary tumors.^31^ Accordingly, as the evidence suggests that increased risk for non‐thyroid SPC could be related to treatment for DTC, more restricted use of RAI therapy and EBRT in selected DTC patients has been suggested recently, notably for younger patients with low‐risk disease.^11^, ^34^

Although diagnosis of an SPC does not appear to affect the initial clinical course of DTC in terms of response to RAI and recurrence‐free survival, it does appear to impact overall survival (OS) and disease‐specific survival. Most DTCs remain low‐risk in the context of MPMNs, but are more likely to become concurrent at more advanced stages.^5^, ^30^, ^35^ The OS of patients with DTC and SPC may be up to 4.4 times less than that of patients without SPC. Usually, patients with DTC and synchronous SPC have worse prognoses in terms of disease stage and mortality than patients with metachronous SPC or without SPC.^14^, ^34^, ^35^ It is currently unclear whether I^131^ or EBRT therapy increases the mortality risk due to SPC.^16^

## 3 TREATMENT RECOMMENDATIONS

Treatment of synchronous MPMN can be a challenge. There are no well‐established, evidence‐based guidelines for this patient group. MPMN also affect enrolment in clinical trials because patients with a prior or current cancer are excluded from most trials. Therefore, for management of synchronous or metachronous MPMN with concurrent active disease, only case reports are published and thus the information given should be taken with caution. Many parameters should be considered (Table 2, ^3^) including malignancy type, disease stage, and overall patient health, leading to individualized treatment in each patient. In this sense, molecular profiling might help to choose the best approach.^3^, ^5^, ^36^, ^37^, ^38^, ^39^

**TABLE 2:** Treatment considerations for patients with MPMN.^3^

Synchronous multiple primaries	Metachronous multiple primaries^a^
Points for consideration when deciding on treatment	
• Malignancy types and each disease stage • The most significant tumor in terms of prognosis ◦ The tumor that is more detrimental to the patient's survival or quality of life ◦ The chance for a curative approach or palliative situation ◦ If the situation is palliative, tumor metastasis, and tumor dynamics (imaging, tumor marker) ◦ Therapeutic options • Local or systemic treatment strategy focus ◦ Radical treatment for one of the synchronous tumors plus sequential treatment for the second malignancy • Anticipated problems • Systemic therapy regimen active for all diagnoses ◦ Potential for interaction between different regimens ◦ Literature about any combination therapy ◦ Evidence the combination can be given ◦ Treating the two malignancies in a cyclical manner • Tumor profiling (e.g. targeted panel sequencing) and the possibility of a common genetic background that enables a common strategy option	• Curative intent for the second primary cancer • Prior treatment for the previous cancer diagnosis ◦ Potential for treatment‐induced second primary • Anticipated complications based on prior primary evolution and previous anticancer therapy • Possible carcinogenic factors that can be managed ◦ Specific treatment • Cancer predisposition for multiple primaries ◦ Predisposition for more cancer that requires screening for prior to initiating treatment

^a^ If the first malignancy is still present, considerations for synchronous multiple primaries apply.

As it is known, in the initial scenario, the therapeutic approach to DTC mainly relies on surgery and RAI with I^131^.^40^ However, up to 20% of DTC patients present at an advanced stage (aDTC) at diagnosis, with distant metastasis and/or locally advanced disease.^41^ Moreover, up to 30% of patients with initial early‐stage disease (eDTC) relapse to an aDTC.^42^ Additionally, one‐third of patients with aDTC at diagnosis and nearly two‐thirds at follow‐up will become refractory to RAI (radioiodine‐refractory DTC; RR‐DTC) during treatment.^43^ As previously described, the OS of DTC patients is high,^6^ but in aDTC the OS decreases markedly, with a 10‐year survival rate for RAI‐responders of 56% compared with 10% for RR‐DTC patients.^44^ Therapy for patients with clinically relevant (symptomatic and/or rapidly progressive) RR‐DTC will involve locoregional techniques and systemic drugs, with the latter mostly based on antiangiogenic multikinase inhibitors (MKIs).^37^, ^40^, ^41^, ^42^, ^43^

Two randomized, placebo‐controlled, multicenter, double‐blind, phase III clinical trials (DECISION, and SELECT) have resulted in US Food and Drug Administration (FDA) and European Medicines Agency (EMA) approval of MKIs (sorafenib and lenvatinib) for treatment of progressive RR‐DTC in adults.^45^, ^46^ More recently, based on results of the phase III COSMIC‐311 trial, the FDA has granted a breakthrough therapy designation for cabozantinib as a possible therapeutic option for patients with RAI‐refractory DTC that has progressed after previous therapy, which is currently pending approval.^47^ These MKIs, however, have also proven useful for the treatment of other advanced cancers. They are currently approved by the FDA and/or EMA for use in unresectable hepatocellular carcinoma (all 3), advanced RCC (all 3, with lenvatinib plus everolimus or pembrolizumab) and advanced endometrial carcinoma (lenvatinib plus pembrolizumab) in patients who have disease progression following previous systemic therapy and who are not medically suitable for curative surgery or radiation. Additionally, other non‐registrational studies have shown the potential efficacy of MKIs alone or given with other drugs in other advanced cancers, thus increasing their potential usefulness in scenarios associated with DTC and MPMN.^48^, ^49^

Nowadays, there are just a few reports of aDTC in the context of MPMN^50^, ^51^, ^52^, ^53^, ^54^ and there is no definitive published clinical evidence supporting the use of MKIs in patients with MPMNs and aDTC. The usefulness of MKIs in patients with MPMNs (including DTC) has been described in some patients though, with comparable results to those in a non‐MPMN context.^53^, ^55^, ^56^, ^57^, ^58^ Despite the lack of sound clinical evidence, there is sufficient pathophysiological rationale to use MKIs, particularly lenvatinib alone or in combination, for specific combinations of MPMNs, including DTC and most common associated SPCs.^48^, ^49^, ^59^ Furthermore, there might be an even greater potential benefit of combining an MKI and a TKI for treatment of advanced malignancies (including DTC) according to therapeutic molecular targets based on common driver gene alterations and/or activated (initial or escape) signaling pathways.^24^, ^32^, ^33^, ^34^, ^35^, ^44^, ^45^, ^59^, ^60^ For example, lenvatinib has shown promise in the simultaneous management of metastatic RR‐DTC and associated bowel malignancies in a patient with familial adenomatous polyposis.^53^ Currently there is an ongoing clinical trial evaluating the usefulness of a therapeutic alternative for MPMN (including DTC) (ClinicalTrials.gov Identifier: NCT04182789. KN035 in Patients With Advanced Multiple Primary Tumors. CPOG035‐01).

To add more complexity to this picture, the use of an MKI even when it is established as standard therapy for a given condition, could be detrimental.^61^, ^62^ For example, imatinib has been associated with increased risk for hematologic malignancies (mostly chronic myeloid leukemia) and worse outcomes than expected in patients with SPCs.^63^, ^64^, ^65^ SPCs have also been described in patients with advanced DTC treated with sorafenib.^45^, ^66^

## 4 DISCUSSION

MPMNs associated with DTC are increasingly frequent. There is scarce definitive scientific evidence for its management to date. Treatment decisions must be individualized, according to the available published literature and the rational basis of the advantages and disadvantages of each therapeutic modality.

## 5 CONCLUSIONS

Although to date we lack specific publications with solid scientific evidence for treatment of aDTC in the context of MPMNs, MKI therapies could be one of the main therapeutic approaches in this scenario, taking into account not only the specific separately reported associated success rates of MKIs (particularly lenvatinb alone or in combination) in some of the major associated cancers and in advanced RR‐DTC, but also the potential implications of the recent advances in the knowledge of specific molecular/genetic markers for each tumor and its immediate consequential potential modifications in the current and near future management. The therapeutic approach to these conditions should always be individualized using tumor board discussion and ensuring multidisciplinary coordinated care, but hopefully forthcoming information based on currently ongoing and future MPMNs clinical trials may help to offer even more personalized and effective single or multimodal treatment alternatives.

## AUTHOR CONTRIBUTION

None.

## FUNDING INFORMATION

The authors received honoraria payment from Eisai Farmacéutica SA in line with ICMJE guidelines.

## CONFLICT OF INTEREST

Dr Sambo Marcel has received research funding, honoraria, and non‐financial or other support from Eisai Farmacéutica SA.

## ETHICS STATEMENT

None.

## Data Availability

None.
